# Supplementary material for: EK100 and Antrodin C Improve Brain Amyloid Pathology in APP/PS1 Transgenic Mice by Promoting Microglial and Perivascular Clearance Pathways
Source: Int J Mol Sci. 2021 Sep 27;22(19):10413. doi: 10.3390/ijms221910413 (PMC8508921; doi:10.3390/ijms221910413)
Supplement: Supplementary file 1 [file ijms-22-10413-s001.zip › ijms-1392930-supplementary.pdf]

Supplementary material

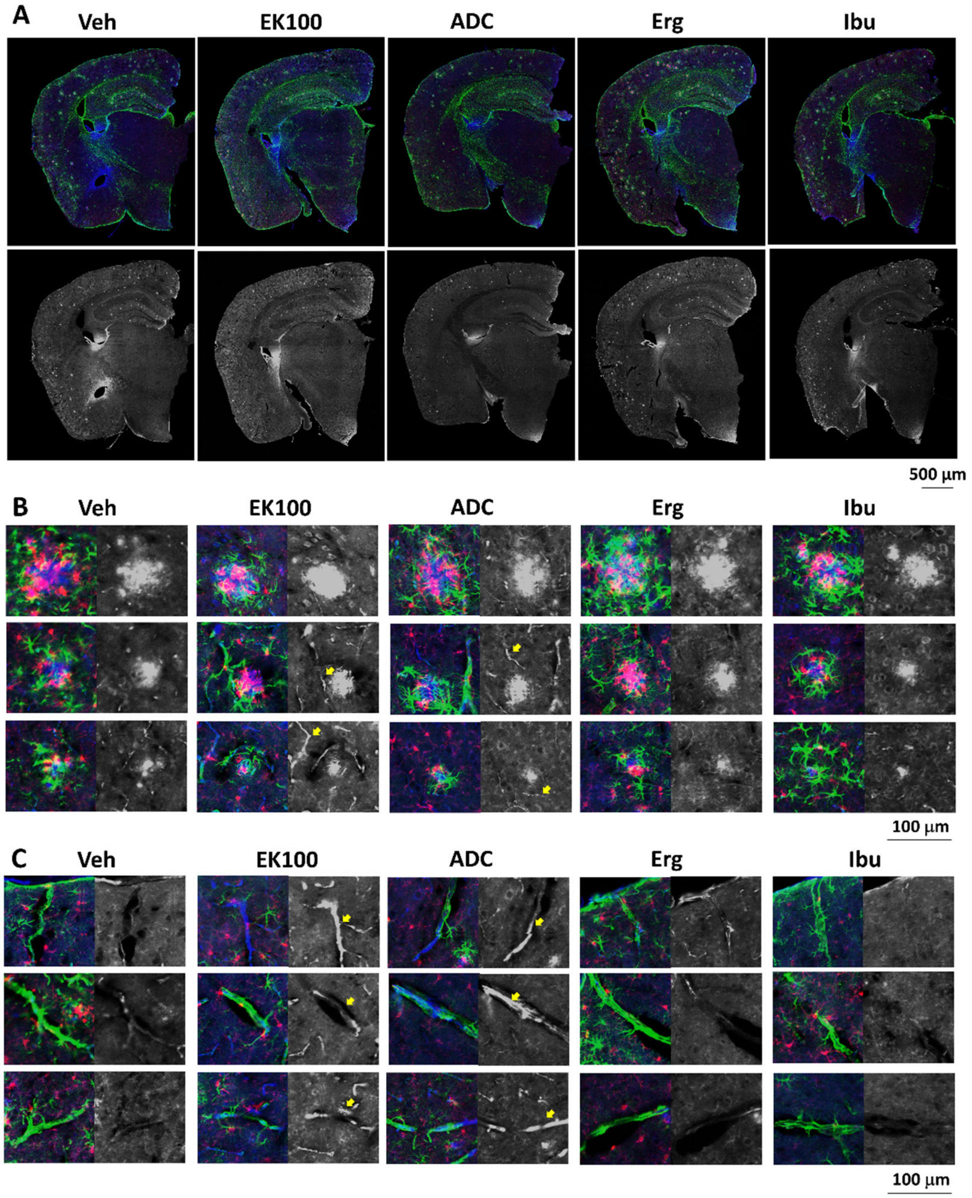

Figure S1. EK100 and antrodin C reduce amyloid plaque burden and plaque-associated glial cluster, and increase perivascular deposition of Aβ in APP/PS1 mice. APP/PS1 transgenic mice orally administered with vehicle (Veh) or EK100, ergosterol (Erg), antrodin C (ADC) and ibuprofen (Ibu) (30 mg/kg/day, n=6 each) for 1 months, and then amyloid plaques and perivascular deposition of Aβ, microglia, and astrocytes were immune-stained with AB10, Iba-1 and GFAP antibodies, respectively. The representative fluorescent images of AB10 (blue in the merged panel), Iba-1 (red in the merged panel) and GFAP (green in the merged panel) in the area including S1,

secondary auditory, and temporal association cortex were shown. Scale bar: 500 μm in panel A and 100 μm in panel B and C.

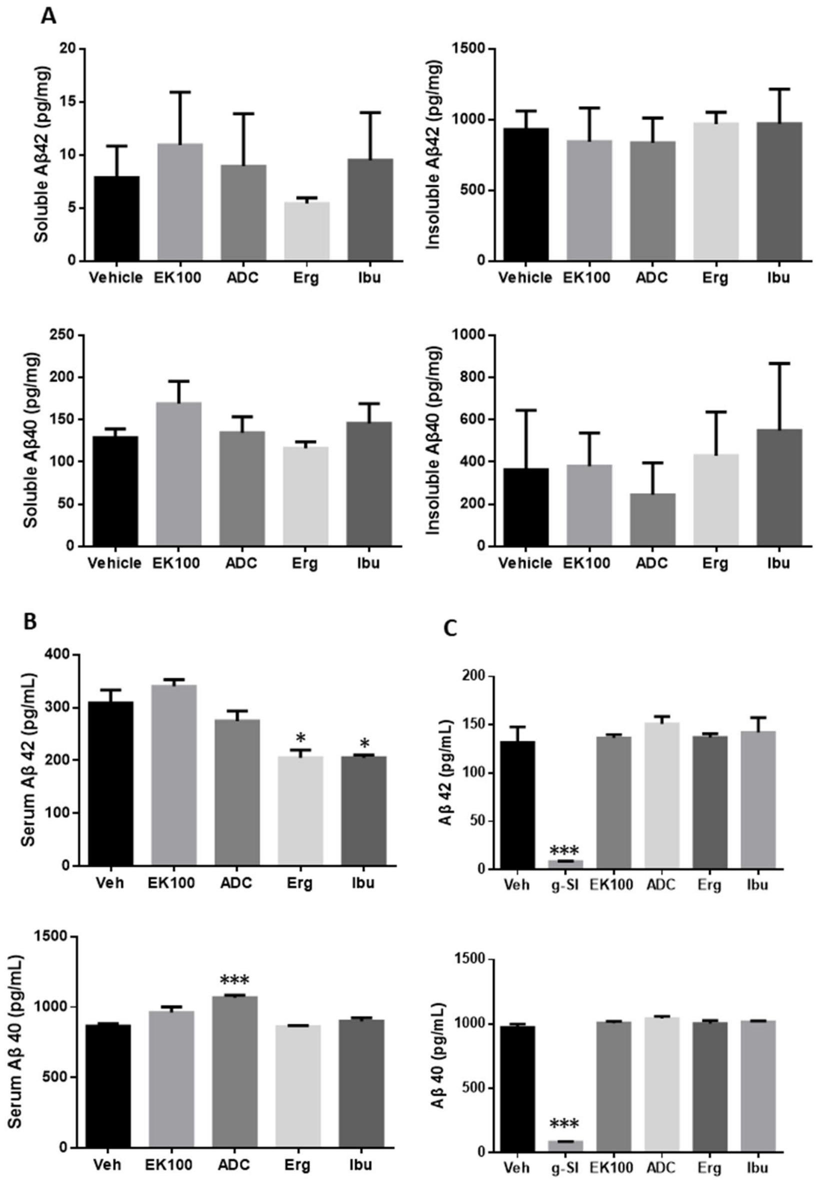

Figure S2. The effects of four reagents on Aβ level in hippocampus and serum of APP/PS1 mice**.** APP/PS1 transgenic mice orally administered with vehicle (Veh) or EK100, ergosterol (Erg), antrodin C (ADC) and ibuprofen (Ibu) (30 mg/kg/day, *n*=6

each) for 1 months, and then the level of Aβ1-40 and Aβ1-42 in hippocampal homogenates (A) and serum (B) were determined by enzyme-linked immunosorbent assay (ELISA). SH-SY5Y-APP695 cells were treated with 50 H-SY5Y-APP695 cells were treated with vehicle (Veh), 1 μM of γ-secreatase inhibitor (g-SI), or 50 μM of EK100, ergosterol (Erg), antrodin C (ADC) and ibuprofen (Ibu) for 24 hr. Aβ1-40 and Aβ1-42 in the conditioned medium were measured by ELISAs. Significant difference between vehicle and treated cells are indicated by ***p<0.001. (n = 4).

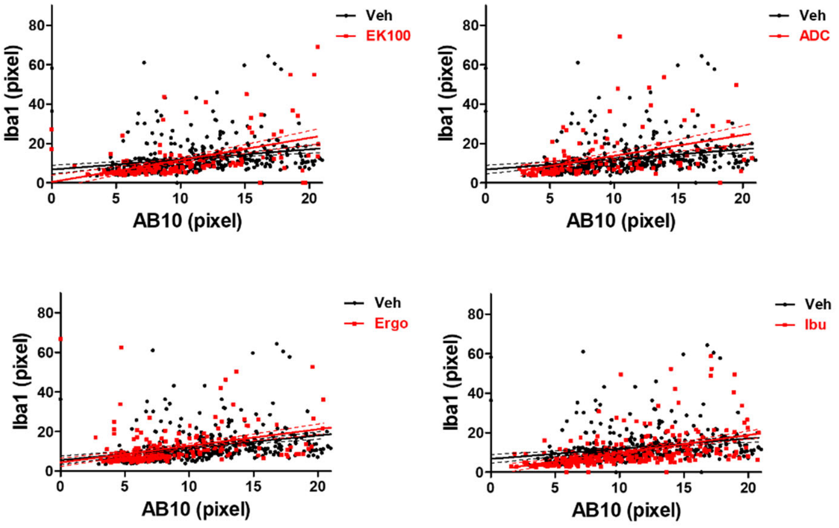

Figure S3. Plaque-associated microglia clustering is not affected by the treatment of four reagents. Scatter plots of AB-10-stained area of each plaque-associated microglia cluster in the brain slice. Solid lines: linear regression; dashed line 95% confidence intervals.
